# Exploring the Nuanced Links Between Internet Use and Subjective Well-Being Among Older Adults: A Nordic Population-Based Study

**DOI:** 10.3389/fpsyg.2021.797269

**Published:** 2022-01-28

**Authors:** Emilia W. E. Viklund, Anna K. Forsman

**Affiliations:** Faculty of Education and Welfare Studies, Health Sciences, Åbo Akademi University, Vaasa, Finland

**Keywords:** subjective well-being, older person, internet use, survey, questioner, Finland, Sweden, older adult

## Abstract

**Aim:**

The aim was to explore the various associations between subjective well-being and internet use among older adults in two regions in Finland and Sweden.

**Methods:**

The data was collected through a population-based survey (*N* = 9,386) as part of the GERDA project conducted in 2016. The connection between subjective well-being (measured by perceived meaningfulness, happiness and life satisfaction) and internet use (distinguishing between internet users, non-users and users with support, and diverse internet activities) was studied by conducting binary regression analyses, calculating odds ratios with 95% confidence intervals. The analyses also controlled for key subjective well-being covariates.

**Results:**

Statistically significant associations were found between perceived life meaningfulness and internet use. When looking into the specific internet-based activities under study, activities related to leisure and entertainment showed statistically significant associations to perceived meaningfulness as well as perceived happiness, also after controlling for potential covariates. However, internet use and the different internet activities failed to show statistical significant associations to life satisfaction in the adjusted regression model.

**Conclusion:**

The things we do on the internet (the activities) as well as how we conceptualize and measure subjective well-being in this type of research studies seem to matter when it comes to the relationship between subjective well-being and internet use in later life. Internet use and internet activities displayed various connections to the subjective well-being proxies used in this study. Therefore, the complexity and multidimensionality of both subjective well-being and internet use and related links need to be carefully explored in order to deepen our understanding of experienced well-being among older adults in a digitized world.

## Introduction

The COVID-19 pandemic, caused by the spread of the infectious disease related to the newly discovered coronavirus, has raised aging and health as well as digital technology on the global agenda. The increased interest in older adults’ health can perhaps be connected to older age (70 +) being one of the risk factors identified by experts for developing severe illness from the virus (World Health Organization, 2021). The virus mitigating measures recommended from the beginning of the pandemic were physical distancing from persons living outside their own household and avoiding public spaces and social gatherings (Finnish Institute for Health and Welfare, 2021). As a consequence, many people have changed the way in which they work, go about their daily business, meet friends and family members as well as participate in hobbies and activities. Hence, digital technology has become a cornerstone in the efforts to tackle the virus while we continue to lead our lives in new ways (European Commission, 2021) using online services more frequently than ever before–or even taking the first few steps into cyber space (The Swedish Internet Foundation, 2020). This especially holds true for older persons as a recent survey shows that the majority of the new internet-users in Sweden in 2020 were persons aged 76 years or older (The Swedish Internet Foundation, 2020). Furthermore, the share of older internet users within the population as a whole has steadily increased in the Nordic region during the last decade (23% in 2010 to 69% among 75 + years in 2019 in Sweden, The Swedish Internet Foundation, 2019), a trend magnitude not seen in Southern and Eastern Europe (Eurostat, 2020). The Nordic countries are being seen as digital forerunners in a European as well as in a global context, currently developing national strategies outlining the delivery and implementation of digital public services, digital security (Randall et al., 2018) and digital health and welfare solutions (Nordic Innovation and Nordic Welfare Centre, 2019). Evidently, digital technology is an essential part of the society–and correspondingly also in our daily lives.

The determinants of subjective well-being have received an increased interest in recent years due to the availability of information from different well-known surveys (Becchetti et al., 2012) and various factors in the social environment are being recognized in many of the models defining health and well-being (e.g., The rainbow model of health by Whitehead and Dahlgren, 2006). Even though digital technology and internet use can be seen as examples of such socio-environmental factors, they are rarely included in the studies of subjective well-being among the general population (Castellacci and Tveito, 2018), not to mention the aging population (Gallistl and Wanka, 2018; Peine and Neven, 2019). However, the current limited evidence base exploring whether and how the use of internet and related digital tools can be connected to well-being in later life, points at advantageous effects, such as lowering levels of loneliness, anomie (Schlomann et al., 2020) and social isolation (Hajek and König, 2019) and enhancing social connectedness (Sinclair and Grieve, 2017) and autonomy (Schlomann et al., 2020), but there are also contradictions, especially when it comes to loneliness (Shah et al., 2021). A theoretical model by Nowland et al. (2018) suggests that when internet is being used for enhancing existing relationships and forming new social contacts it can reduce loneliness, whereas the opposite effect might be observed if digital tools totally replace offline social activities (Nowland et al., 2018). The connection between internet use and different forms of loneliness is therefore argued to be dependent on the way in which internet and related digital tools are being used (Szabo et al., 2019). Furthermore, Lifshitz et al. (2018) looked into the connection of different internet activities to subjective well-being in later life and found that after adding sociodemographic variables into the analysis, only online leisure activities predicted subjective well-being by increasing life satisfaction and decreasing depression levels. Hence, internet use *per se* may not be connected to well-being in later life–there is a need to explore the connection between different kinds of internet activities and the subjective well-being of older adults.

Besides the importance of exploring internet use in terms of activities or content, there seems to be an additional element that needs to be taken into consideration when exploring the complex link between well-being and internet use in later life. A previous study by Nordmyr and Forsman (2016) found that internet use among older adults seems to be connected to emotional aspects of health–but not to the physical or social components. Similarly, Quintana et al. (2018) suggest that internet use has a positive impact on evaluative and eudaimonic dimensions of subjective well-being in later life, while the connection toward hedonic dimensions seems to be more limited. Thus, the scarce but growing literature base suggests that internet use does not seem to be connected to all of the dimensions of older adults’ health or subjective well-being. This requires further investigation.

The question of what constitutes our well-being and characterizes a good life has engaged scholars from a wide-range of disciplines and the literature showcases several attempts to conceptualize the well-being concept (Dodge et al., 2012). However, a traditional and widely used way to define the concept of well-being is by distinguishing between hedonic and eudaimonic approaches (Ryan and Deci, 2001). The hedonic approach generally emphasizes pleasure, enjoyment and the absence of discomfort in the search for well-being (Huta and Waterman, 2014), whereas the eudaimonic perspective advocates for the inclusion of aspects related to positive functioning and flourishing for understanding the concept (Huta and Waterman, 2014). Today, scholars seem to agree upon the multidimensional notion of well-being (Keyes, 2006; Vanhoutte, 2014)–a combination of hedonic and eduamonic approaches are requested to reach a more full picture (Ryan and Deci, 2001; Huta and Ryan, 2010). Moreover, in the current interdisciplinary field of research connected to well-being, numerous terms and concepts (e.g., happiness, mental well-being, psychological well-being, subjective well-being and positive mental health, to mention only a few) referring to the positive and subjective dimensions of our psychosocial health are circulating, which complicates the exploration of the literature and comparison of studies. Subjective well-being is, however, the most frequently used conceptualization (Martela and Sheldon, 2019) and in a review study on subjective well-being, health and aging, Steptoe et al. (2015) distinguished between three dimensions of subjective well-being: hedonic (happiness and everyday life feelings), evaluative (life satisfaction), and eudaimonic (positive functioning and meaning or purpose in life). Each understood to capture different nuances of the concept (Steptoe et al., 2015).

In a previous study (Viklund et al., 2021) we explored the association between perceived meaningfulness and internet use in later life among older adults in Finland and Sweden. The findings from this study indicated statistically significant positive associations between the measured component of eudaimonic subjective well-being (i.e., perceived meaningfulness) and internet use–both when looking at general use and at internet activities related to hobbies and entertainment in particular. Further, based on findings presented by other studies, internet use as such might not be connected to subjective well-being *per se*. Hence, the complexity and multidimensionality of both subjective well-being and internet use and related links need to be carefully explored in order to deepen our understanding of experienced well-being among older adults in a digitized world. Therefore, in this study we want to build on to our previous work and the growing literature base by simultaneously exploring three proxies all representing different dimensions of subjective well-being, in relation to internet use and internet activities in later life.

## Aim

The aim of the study was to explore the various associations between subjective well-being (using three proxies for subjective well-being: meaningfulness, happiness and life satisfaction) and internet use among older adults in two regions in Finland and Sweden. Further, the statistical analyses adjusted for key subjective well-being covariates.

## Materials and Methods

### Study Design and Data Material

A population-based cross-sectional survey study was conducted in 2016 in Västerbotten (Sweden) and in Österbotten (Finland), as a part of the GErontological Regional DAtabase and Resource Centre (GERDA) project. The aim of the GERDA project was to gather information on older adults’ health and living conditions. The survey was posted to all individuals born in 1950, 1945, 1940, 1935, and 1930, living in rural areas and the city of Seinäjoki (Finland), to every second person in the city of Vaasa (Finland) and to every third person within the same age cohorts in the cities of Umeå (Sweden) and Skellefteå (Sweden) (in total 14,805 persons). The population register was obtained from the National Tax Board in Sweden and the Population Register Centre in Finland (for further details, see the GERDA Method Report written for the study in GERDA, 2011). In total, 9,386 individuals participated in the survey study, giving a response rate of 63.40 per cent. The total number of respondents per outcome variable were 9,175 (Meaningfulness), 9,221 (Happiness) and 9,082 (Life satisfaction). The variables included in the analysis were controlled for missing data. For the outcome variables the missing values were *N* = 211 (2.2%) for Meaningfulness, *N* = 165 (1.8%) for Happiness and *N* = 302 (3.2%) for Life satisfaction. For the variables measuring individual characteristics, socio-environmental factors, internet use and self-rated health status, the missing values ranged from *N* = 0 for study regions to *N* = 437 (4.7%) for internet use.

### Measurements and Variables

The outcome variables in the statistical analyses in this study are three separate variables assumed to measure different dimensions of subjective well-being.

### Perceived Meaningfulness, Happiness and Life Satisfaction as Proxies for Subjective Well-Being

Perceived meaningfulness in life is an important aspect of subjective well-being and is generally connected to the eudaimonic dimensions of well-being. Experienced meaningfulness can be about life being comprehensible (coherence), having a direction or life goals (purpose) or about a full life–having a life worth living (significance) (Martela and Steger, 2016). Compared to measurements of other dimensions of subjective well-being such as life satisfaction, there is a general lack of instruments for measuring meaning in life (Forgeard et al., 2011). Meaning in life is in this study measured with a one-item question (“How meaningful do you perceive your life at the moment?”) with five answering options ranging from very meaningful to very meaningless. The question was dichotomized for the binary regression analyses.

Happiness is commonly understood as the affective evaluation of one’s life situation and relates to the dominance of positive affect over negative affect in one’s life (Diener, 1984). Happiness in this sense refers to episodic happiness–or feeling happy in the present moment (Abdel-Khalek, 2006). However, happiness is argued to be a vague construct and many scholars have therefore moved away from the concept or added dimensions to the concept while studying it (Forgeard et al., 2011). Life satisfaction is an example of such a dimension that is commonly added to instruments measuring happiness, especially in psychology. However, there are also arguments for keeping the two concepts separated from each other (Huta and Waterman, 2014). In this study happiness is related to the hedonic dimension of subjective well-being and in previous studies both single-item scales and multiple item-scales are used for measuring happiness (Abdel-Khalek, 2006). Here happiness is assessed by responding to a one-item question (“How happy or unhappy do you feel at the moment?”) with five answering options ranging from very happy to very unhappy. The question was dichotomized for the binary regression analyses.

Life satisfaction is the most commonly used proxy for subjective well-being (Forgeard et al., 2011; Steptoe and Fancourt, 2019; Ramia and Voicu, 2020) and is often assessed by self-evaluations of one’s general life situation (Forgeard et al., 2011) or focusing on specific life domains (Pavot and Diener, 2008). Life satisfaction is related to the affective dimensions of subjective well-being (such as happiness), but also partially independent of it and is thereby sometimes seen as a separate dimension (Pavot and Diener, 2008). The life satisfaction variable in this study is based on a question included in a shorter version of the Positive Life Orientation Scale (“Are you satisfied with your life?”) and is seen as related to the evaluative dimension of subjective well-being. The survey question had two answering options, yes or no.

### Key Subjective Well-Being Covariates

It is important to recognize that certain factors associated to individual characteristics and our social environment make it easier to experience well-being across the lifespan.

Internet use and/or engagement in specific internet-based activities can be seen as such socio-environmental factors in an increasingly digitized daily life. In this study internet use is measured by variables based on two different survey questions, one dichotomized variable measuring internet use by separating independent internet users from persons using internet with the support of others, and non-users and by five variables created from a multiple-answer question measuring recent internet-based activities. These variables were also recoded into dummy variables and treated as separate categorical variables in the statistical analyses. The internet activities are: Instrumental use (bank, travel arrangements, social security), Informational use (newspapers, news forums), Leisure/entertainment (music, movies, games, forums connected to interests), Social networking and support (Skype, Facebook, online dating, and email) and Other activities (blogs, sports, and church services).

In addition to internet use, there are also several other socio-environmental factors associated with subjective well-being. Previous studies have highlighted that well-being is not equally distributed among people–individual and socio-environmental characteristics influence the distribution of population well-being and its various dimensions (Deeming, 2013). For instance, happiness is suggested to be influenced by age, ethnicity, health status, education and household composition (Deeming, 2013). Lyubomirsky et al. (2005) also relates income and marital status to happiness. For life satisfaction economic circumstances such as income level as well as age, also seem to matter (Deeming, 2013; Steptoe and Lassale, 2018), but not education and ethnicity (Deeming, 2013). Additionally, social activity and engagement appear to have a crucial role for life satisfaction in later life (Steptoe and Lassale, 2018). Similar determinants are also connected to meaningfulness, for example, couples tend to describe their lives as more worthwhile than single people living alone do (Deeming, 2013). However, gender seems to matter when it comes to meaningfulness (women more likely to report a meaningful life), but not to happiness and life satisfaction (Deeming, 2013). A set of variables measuring individual characteristics (gender, age) and socio-environmental factors (study region, educational level, income level, marital status) are therefore included in the analyses related to this study. These variables are recoded, and the cut-off points for the recoding process are chosen in accordance with other scientific articles based on the GERDA study (e.g., Forsman et al., 2012; Viklund et al., 2021).

Health status is another important factor related to subjective well-being and needs to be taken into consideration, especially in studies concerning older adults’ subjective well-being (Deeming, 2013; Steptoe et al., 2015). For instance, chronic diseases seem to diminish hedonic and eudaimonic dimensions of subjective well-being (Steptoe et al., 2015) and better self-rated health has been linked to experiencing meaningfulness (Steptoe and Fancourt, 2019; Tiilikainen et al., 2021). The analyses in this study also included a variable measuring self-rated health.

All the included variables are displayed in Tables 1–3 below.

**TABLE 1:** The distribution of perceived meaningfulness among all the included variables and results from Person’s chi-square test presenting between-group comparison of perceived meaningfulness.

	Perceived meaningfulness, no	Perceived meaningfulness yes	All	*X* ^2^
**Internet use**				
Not using internet	866 (30.2)	2005 (69.8)	2871 (32.8)	*p* ≤ 0.001
Internet users with support	107 (17.2)	516 (82.8)	623 (7.1)	
Independent internet users	732 (13.9)	4532 (86.1)	5264 (60.1)	
**Internet activities**				
Instrumental use	694 (14.1)	4226 (85.9)	4920 (53.6)	*p* ≤ 0.001
Informational use	677 (14.0)	4167 (86.0)	4844 (52.8)	*p* ≤ 0.001
Leisure/entertainment	383 (12.7)	2639 (87.3)	3022 (32.9)	*p* ≤ 0.001
Social network and support	499 (13.1)	3300 (86.9)	3799 (41.4)	*p* ≤ 0.001
Other activities	42 (13.7)	264 (86.3)	306 (3.3)	*p* = 0.006
**Gender**				
Man	847 (20.1)	3377 (79.9)	4224 (46.1)	*p* = 764
Woman	982 (19.8)	3966 (80.2)	4948 (53.9)	
**Age**				
65	338 (12.4)	2379 (87.6)	2717 (29.7)	*p* ≤ 0.001
70	457 (16.3)	2349 (83.7)	2806 (30.7)	
75	363 (21.9)	1291 (78.1)	1654 (18.1)	
80	362 (28.7)	899 (71.3)	1261 (13.8)	
85	308 (43.0)	408 (57.0)	716 (7.8)	
**Study region**				
Finland	977 (19.9)	3943 (80.1)	4920 (53.6)	*p* = 0.821
Sweden	853 (20.0)	3402 (80.0)	4255 (46.4)	
**Marital status**				
Single	783 (32.7)	1613 (67.3)	2396 (26.4)	*p* ≤ 0.001
In a relationship	1031 (15.4)	5663 (84.6)	6694 (73.6)	
**Educational level**				
Low	843 (25.1)	2515 (74.9)	3358 (37.1)	*p* ≤ 0.001
Middle	625 (18.7)	2843 (81.3)	3495 (38.6)	
High	307 (13.9)	1894 (86.1)	2201 (24.3)	
**Income level**				
Low	598 (26.3)	1677 (73.7)	2275 (25.6)	*p* ≤ 0.001
Middle	825 (20.0)	3310 (80.0)	4135 (46.5)	
High	340 (13.7)	2143 (86.3)	2483 (27.9)	
**Self-rated health**				
Moderate/poor	1162 (35.4)	2122 (64.6)	3284 (36.1)	*p* ≤ 0.001
Good	434 (14.8)	2496 (85.2)	2930 (32.2)	
Very good	208 (7.2)	2681 (92.8)	2889 (31.7)	

**TABLE 2:** The distribution of perceived happiness among all the included variables and results from Person’s chi-square test presenting between-group comparison of perceived happiness.

	Perceived happiness, no	Perceived happiness, yes	All	*X* ^2^
**Internet use**				
Not using internet	820 (28.5)	2054 (71.5)	2874 (32.6)	*p* ≤ 0.001
Internet users with support	142 (22.6)	486 (77.4)	628 (7.1)	
Independent internet users	884 (16.7)	4420 (83.3)	5304 (60.2)	
**Online activities**				
Instrumental use	853 (17.2)	4102 (82.8)	4955 (53.7)	*p* ≤ 0.001
Informational use	813 (16.7)	4060 (83.3)	4873 (52.8)	*p* ≤ 0.001
Leisure/entertainment	480 (15.8)	2557 (84.2)	3037 (32.9)	*p* ≤ 0.001
Social network and support	629 (16.4)	3197 (83.6)	3826 (41.5)	*p* ≤ 0.001
Other activities	65 (21.2)	242 (78.8)	307 (3.3)	*p* = 0.971
**Gender**				
Man	923 (21.7)	3334 (78.3)	4257 (46.2)	*p* = 0.350
Woman	1036 (20.9)	3925 (79.1)	4961 (53.8)	
**Age**				
65	436 (16.0)	2295 (84.0)	2731 (29.7)	*p* ≤ 0.001
70	544 (19.2)	2289 (80.8)	2833 (30.8)	
75	370 (22.3)	1288 (77.7)	1658 (18.0)	
80	339 (26.9)	919 (73.1)	1258 (13.7)	
85	266 (36.9)	454 (63.1)	720 (7.8)	
**Study region**				
Sweden	1016 (23.6)	3280 (76.4)	4296 (46.6)	*p* ≤ 0.001
Finland	944 (19.2)	3981 (80.8)	4925 (53.4)	
**Marital status**				
Single	807 (33.5)	1599 (66.5)	2406 (26.3)	*p* ≤ 0.001
In a relationship	1134 (16.8)	5598 (83.2)	6732 (73.7)	
**Educational level**				
Low	836 (24.7)	2544 (75.3)	3380 (37.2)	*p* ≤ 0.001
Middle	733 (20.9)	2778 (79.1)	3511 (38.6)	
High	361 (16.4)	1844 (83.6)	2205 (24.2)	
**Income level**				
Low	606 (26.5)	1680 (73.5)	2286 (25.6)	*p* ≤ 0.001
Middle	927 (22.3)	3224 (77.7)	4151 (46.5)	
High	355 (14.2)	2143 (85.8)	2498 (28.0)	
**Self-rated health**				
Moderate/poor	1184 (35.9)	2116 (64.1)	3300 (36.1)	*p* ≤ 0.001
Good	509 (17.3)	2433 (82.7)	2942 (32.1)	
Very good	236 (8.1)	2674 (91.9)	2910 (31.8)	

**TABLE 3:** The distribution of perceived satisfaction with life among all the included variables and results from Person’s chi-square test presenting between-group comparison of perceived satisfaction with life.

	Perceived satisfaction with life, no	Perceived satisfaction with life, yes	All	*X* ^2^
**Internet use**				
Not using internet	198 (7.1)	2609 (92.9)	2807 (32.3)	*p* ≤ 0.001
Internet users with support^..^	37 (6.0)	580 (94.0)	617 (7.1)	
Independent internet users	240 (4.6)	5016 (95.4)	5256 (60.6)	
**Online activities**				
Instrumental use	229 (4.7)	4684 (95.3)	4913 (54.1)	*p* = 0.002
Informational use	211 (4.4)	4625 (95.6)	4836 (53.2)	*p* ≤ 0.001
Leisure/entertainment	139 (4.6)	2867 (95.4)	3006 (33.1)	*p* = 0.031
Social network and support	177 (4.7)	3608 (95.3)	3785 (41.7)	*p* = 0.016
Other activities	16 (5.3)	287 (94.7)	303 (3.3)	*p* = 0.956
**Gender**				
Man	222 (5.3)	3986 (94.7)	4208 (46.3)	*p* = . 764
Woman	264 (5.4)	4609 (53.6)	4873 (53.7)	
**Age**				
65	152 (5.6)	2553 (94.4)	2705 (29.8)	*p* = 0.109
70	137 (4.9)	2671 (95.1)	2808 (31.0)	
75	94 (5.7)	1546 (94.3)	1640 (18.1)	
80	54 (4.4)	1165 (95.6)	1219 (13.4)	
85	48 (6.9)	644 (7.1)	692 (7.6)	
**Study region**				
Sweden	247 (5.8)	3982 (94.2)	4229 (46.6)	*p* = 0.052
Finland	239 (4.9)	4616 (95.1)	4855 (53.4)	
**Marital status**				
Single	193 (8.2)	2160 (91.8)	2353 (26.1)	*p* ≤ 0.001
In a relationship	290 (4.4)	6360 (95.6)	6650 (73.9)	
**Educational level**				
Low	189 (5.7)	3130 (94.3)	3319 (37.0)	*p* = 0.547
Middle	179 (5.2)	3280 (94.8)	3459 (38.6)	
High	112 (5.1)	2074 (94.9)	2186 (24.4)	
**Income level**				
Low	172 (7.7)	2054 (92.3)	2226 (25.3)	*p* ≤ 0.001
Middle	225 (5.5)	3869 (94.5)	4094 (46.5)	
High	77 (3.1)	2416 (96.9)	2493 (28.3)	
**Self-rated health**				
Moderate/poor	374 (11.7)	2823 (88.3)	3197 (35.4)	*p* ≤ 0.001
Good	72 (2.5)	2846 (97.5)	2918 (32.4)	
Very good	36 (1.2)	2869 (98.8)	2905 (32.2)	

## Ethics

The survey data collection that the study at hand is based upon were reviewed and approved by the Regional Ethical Review Board in Umeå, Sweden (05/084 and 2016/367- 1212 32). In Finland, ethical approval is not required for anonymous postal surveys.

## Analyses

The software SPSS version 27 (IBM Corp, Armonk, NY, United States) was used for the statistical analyses. The distribution of each of the different dimensions of subjective well-being among the individual characteristics, socio-environmental variables, self-rated health status and internet-related variables are presented in Tables 1–3. In order to explore the between-group comparison of each of the three aspects of subjective well-being in relation to internet activities, individual characteristics, socio-environmental variables and health status, Pearson’s chi-square tests were conducted (Tables 1–3). Further, three logistic regression analyses were conducted to explore the association between internet activities and each of the three aspects of subjective well-being. Variables measuring individual characteristics, socio-environmental factors and self-rated health were added to the logistic regression analyses in order to control for potential covariates in the adjusted model for each of the three outcome variables. Odds ratios (ORs) with 95% confidence intervals were calculated and are presented in Table 4. The odds ratios (OR) presented in the first column represent the likelihood of perceiving meaningfulness, happiness and life satisfaction among non-internet users, persons using internet with support, independent internet users and among internet users, using different kinds of internet activities. The OR presented in the second column of the Table 4, represents the likelihood of perceiving meaningfulness, happiness and life satisfaction among the variables measuring internet use and internet activities while adjusting for potential covariates among the individual characteristics, socio-environmental and health status variables presented in Tables 1–3. Additionally, the goodness of fit was tested using Nagelkarke R2.

**TABLE 4:** Odds ratio and their 95% confidence intervals of perceived meaningfulness, happiness and life satisfaction as well as the results from Nagelkarke *R*^2^ analysis.

	OR (95% CI)*^a^*	Adj OR (95% CI)*^b^*
**Meaningfulness**		
**Internet use**		
No internet	1.00	1.00
Internet use with support	2.083 (1.667–2.603)***	1.418 (1.104–1.821)*
Independent internet use	2.674 (2.392–2.990)***	1.273 (1.100–1.474)***
Nagelkerke *R*^2^	0.54	0.199
**Internet activities**		
Instrumental use	1.417 (1.229–1.635)***	1.009 (0.858–1.187)
Informational use	1.353 (1.163–1.574)***	1.004 (0.849–1.188)
Leisure/entertainment	1.301 (1.121–1.511)***	1.192 (1.015–1.401)*
Social network and support	1.307 (1.126–1.517)***	1.173 (0.992–1.382)
Other activities	1.294 (0.926–1.807)	1.436 (0.992–2.078)
Nagelkerke *R*^2^	0.053	0.202
**Happiness**		
**Internet use**		
No internet	1.00	1.00
Internet use with support	1.366 (1.114–1.675)*	0.959 (0.755–1.194)
Independent internet use	1.996 (1.791–2.225)***	1.103 (0.956–1.274)
Nagelkerke *R*^2^	0.027	0.175
**Internet activities**		
Instrumental use	1.198 (1.044–1.375)*	0.917 (0.785–1.072)
Informational use	1.350 (1.168–1.5611)***	1.077 (0.917–1.265)
Leisure/entertainment	1.203 (1.047–1.382)*	1.171 (1.007–1.361)*
Social network and support	1.156 (1.004–1.330)*	1.026 (0.879–1.198)
Other activities	0.867 (0.654–1.149)	0.844 (0.619–1.151)
Nagelkerke *R*^2^	0.026	0.176
**Life satisfaction**		
**Internet use**		
No internet	1.00	1.00
Internet use with support	1.190 (0.828–1.709)	0.890 (0.601–1.317)
Independent internet use	1.586 (1.306–1.926)***	0.994 (0.776–1.274)
Nagelkarke *R*^2^	0.007	0.161
**Internet activities**		
Instrumental use	1.083 (0.842–1.394)	902 (0.687–1.184)
Informational use	1.514 (1.160–1.975)*	1.240 (0.936–1.643)
Leisure/entertainment	0.968 (0.753–1.244)	0.911 (0.698–1.189)
Social network and support	0.950 (0.734–1.229)	0.924 (0.703–1.214)
Other activities	0.930 (0.556–1.556)	0.968 (0.557–1.682)
Nagelkerke *R*^2^	0.007	0.159

*^a^OR, odds ratio. CI, confidence interval.*

*^b^OR adjusted for: Age, gender, marital status, study regions, educational level, income level and self-rated health.*

*p < .o5, and ***p ≤ 0.001.

## Results

### Descriptive Statistics

In total, 46.6 per cent of the participants were from Sweden and 53.4 per cent from Finland. The sample had a higher representation of women and the vast majority of the study participants were in a relationship when the study was conducted. About 59 per cent of the whole study sample were internet users (used internet independently) and the most frequently used internet activities were related to practicalities of daily life (instrumental use) and information. Almost 64 per cent had used internet for social networking and support during last month and about 50 per cent for hobbies and entertainment. Only a few (5.2%) of the study respondents indicated that they used internet for other purposes (other activities). However, the internet activities were based on a multiple answer question, meaning that the older adults used internet for more than one purpose at a time. More descriptive statistics of the study sample is presented elsewhere (Viklund et al., 2021).

### The Association Between Subjective Well-Being, Internet Use and Internet Activities

Overall, 80.1 per cent of the total study sample perceived their lives as meaningful, 78.7 per cent felt happy at the moment and 94.6 per cent reported being satisfied with their lives. The results from the Pearson’s chi square tests indicate variance in the association between the three outcome variables and the internet variables. For example, significant between-group differences were found for experiencing meaningfulness in life among all of the variables studying the internet activities (Table 1). For the other two outcome variables, perceived happiness (Table 2) and life satisfaction (Table 3), the variable “Other activities” failed to show statistical significance, whereas all of the other internet-activities showed statistical significance. Further, the individual characteristics, socio-environmental and health status variables included in the analysis also revealed varying between-groups differences. For happiness and meaningfulness, statistical significant differences were found for all of the individual characteristics, socio-environmental and health status variables except for gender (happiness and meaningfulness) and study regions (meaningfulness). When it comes to the last subjective well-being proxy, life satisfaction, only marital status, income level and self-rated health displayed statistical significant differences.

The results regarding meaningfulness from the logistic regression analyses indicate that the older adults who used internet with support and independently have higher odds for perceiving life as meaningful, compared to the older adults who reported that they did not use the internet at all (Table 4). The association between independent and supported internet use and meaningfulness was statistically significant. This also holds true after adjusting for individual characteristics, socio-environmental variables and self-rated health status (Table 4 and column 2). Looking into the specific internet activities and their association to meaningfulness, all of the internet activities, except for “Other activities,” displayed statistically significant associations. The likelihood of perceiving life as meaningful was higher amongst the older adults who used the internet for information, instrumental use, leisure/entertainment and social network and support, compared to those not using these functions. However, after adjusting for potential covariates, only leisure/entertainment related internet activities have a statistically significant association to meaningfulness.

The analysis exploring the association between perceived happiness and internet use points at similar results as the ones for meaningfulness, since the association between internet usage (both with support and independent usage) and happiness was statistically significant (Table 4, column 1). Nonetheless, when individual characteristics, socio-environmental factors and the variables measuring health status were added to the analysis, overall internet use failed to show statistically significant associations to perceived happiness (Table 4, column 2). The odds for feeling happy were lower among the internet users with support compared to the non-users. Moreover, all of the internet activities, except for “Other activities,” displayed statistically significant associations to perceived happiness (Table 4, column 1). When adding potential covariates to the analysis only leisure/entertainment displayed a statistically significant association. Hence, the odds for perceiving happiness in later life increased especially among the onliners, using internet for activities related to leisure and entertainment (Table 4, column 2).

The findings related to perceived life satisfaction and internet use in later life indicate that only independent internet use has a statistically significant association to life satisfaction (Table 4, column 1), but failed to show such association in the second adjusted step (Table 4, column 2). The results concerning internet activities suggest that only informational use was significantly connected to life satisfaction, however, no association was displayed after adjusting the OR for individual characteristics, socio-environmental variables and self-rated health status. It appears that all of the internet activities, except for informational use, had slightly lower odds for being satisfied with later life. Again, several of the socio-environmental variables, individual characteristics and self-rated health seem to be significant covariates to the internet activities when it comes to life satisfaction.

The goodness of fit of the different models is reported by the Nagelkarke’s R-square (Field, 2013) in Table 4.

## Discussion

### Study Findings

This study adds to the growing field of research exploring whether and how socio-environmental factors such as internet use might influence subjective well-being in later life. Unlike many other studies examining the association between older adults’ subjective well-being and internet use, the study at hand explores three different dimensions of subjective well-being separately along with internet use and internet activities, while adjusting for individual characteristics, socio-environmental factors and self-rated health status. The results suggest that both internet activities and different dimensions of subjective well-being should be considered when analyzing the association between subjective well-being and internet use in later life as they display diverse outcomes. However, the study has methodological limitations that should be taken into consideration when interpreting the study findings and will be further discussed in the strengths and limitations section.

Between-group comparisons in the study at hand revealed that after adjusting for potential covariates among variables measuring individual characteristics, socio-environmental factors and health status, internet use overall seems to be statistically significant only in the association to perceived meaningfulness, not to happiness nor life satisfaction. The results regarding the connection between internet use, internet activities and perceived meaningfulness in later life have previously been published (Viklund et al., 2021). However, the study at hand advances the previous study results by simultaneously exploring two other dimensions of subjective well-being in addition to perceived meaningfulness. Quintana et al. (2018) similarly studied older adults’ internet use in relation to the different dimensions of subjective well-being by using longitudinal data. They likewise found a positive and statistical significant connection between internet use and the eudaimonic dimensions of subjective well-being, but not to the hedonic and evaluative dimensions, after adjusting for health and socioeconomic indicators. However, Quintana et al. (2018) did not look into the specific internet activities–they measured internet use with a dichotomous variable created from a question about using the internet/email or not (yes/no). When looking into the specific internet-based activities in this study, activities connected to leisure and entertainment showed statistical significance to perceived meaningfulness and happiness in the adjusted logistic regression models. Similar tendencies have also been presented in previous studies. For example, Lifshitz et al. (2018) described that internet use for leisure activities contributed to increased life satisfaction and decreased depression levels and online hobbies and entertainment proved to be significantly associated to emotional health status among older adults in a study by Nordmyr and Forsman (2016). Thus, the connection between subjective well-being, internet use and related activities seems to differ depending on which proxy is studied. Based on our study findings, we ask ourselves why meaningfulness stands out among the three explored subjective well-being proxies by being statistically significantly connected to overall internet use? And why do activities connected to leisure and entertainment increase the odds for both perceiving life as meaningful and experiencing happiness? Moreover, why do older internet users seem to have lower odds for life satisfaction, compared to the non-users? We reviewed the literature on meaningfulness, happiness and life satisfaction as well as on internet use in an attempt to disentangle these questions.

Vittersø and Søholt (2011) suggested a functional model of well-being, where satisfaction and pleasure are seen as elements of hedonic well-being and engagement and interest are connected to the eudaimonic well-being dimensions. Baumeister et al. (2013) also explored the concepts of happiness and meaningfulness and found some key differences between the concepts. One of these differences is that activities that express and reflect one’s personal preferences are argued to be important for perceiving life as meaningful, whereas they seem to be irrelevant to happiness (Baumeister et al., 2013). A recent Finnish interview study also makes the connection between activities and experienced meaningfulness (Tiilikainen et al., 2021). Tiilikainen et al. (2021) found that different kinds of activities such as daily chores, social contacts and outdoor activities contributed to experiencing daily life as meaningful during the COVID-19 pandemic. Another study (Nordmyr et al., 2020) conducted in a Finnish context, utilizing qualitative focus group methodology, similarly highlights the importance of being able to engage in activities of one’s own choice and having the physical health to pursue activities that give enjoyment and a sense of meaning for experiencing well-being in later life. Apart from the functional ability and capability, previous studies emphasize that social and everyday activities are equally important when it comes to experiencing meaningfulness and well-being (Nordmyr et al., 2020; Tiilikainen et al., 2021). These findings are also in line with the model of healthy aging presented by Bryant et al. (2001) and theories of aging such as the activity theory (Havighurst and Albrecht, 1953).

Based on these previous studies and theoretical perspectives engaging in activities of one’s own choice–activities that give persons enjoyment and a sense of meaning–can in turn contribute to finding life meaningful and experiencing well-being. Such meaningful activities can also encompass online activities. For instance, in a study by Rønning and Sølvberg (2017) older adults described internet use for leisure activities as rewarding and as something that enriched their daily lives. Unlike online banking or eHealth services, older adults themselves make their own choices to engage in internet activities connected to leisure–free from social pressure. Therefore, one possible link between perceived meaningfulness and internet use could be based on the assumption that internet use provides opportunities for older adults to participate in activities they find meaningful. Specifically, the internet could be seen as a tool, facilitating engagement in meaningful activities and participating in meaningful activities could, in turn, contribute to perceiving life as meaningful. Apart from being perceived as meaningful online activities connected to entertainment and leisure might give rise to feelings of pleasure, which perhaps could shed light on the suggested connection to perceived happiness. However, more studies exploring if and how the internet has worked as a tool for facilitating participation in leisure activities during times of isolation due to the COVID-19 pandemic and the relation to subjective well-being would be of interest in order to understand more about the suggested link.

The evaluative dimension, in this study measured by life satisfaction, seems to differ from the other subjective well-being dimensions–and especially from the eudaimonic dimension measured by perceived meaningfulness–when it comes to the association to internet use. When adjusting for the variables measuring individual characteristics, socio-environmental factors and health status in the regression analysis, independent internet users and internet users with support seem to have a tendency to exhibit lower odds for endorsing complete and total life satisfaction, compared to the non-users, even though not statistically significant. Additionally, all of the internet activities, except for informational use seemed to reduce the odds for life satisfaction in the adjusted model. This tendency found in the data, however not statistically significant, contradicts the results presented by previous studies exploring internet use in relation to life satisfaction–where internet use related to online shopping (Lissitsa and Chachashvili-Bolotin, 2016), leisure (Lifshitz et al., 2018), and social network sites (Gaia et al., 2021) increased life satisfaction. The lower odds for life satisfaction among the internet users compared to the non-users seem to be related to the variables controlled for in the adjusted model of the analysis. However, the study findings displayed significant covariates among the indicators for individual characteristics, socio-environmental factors and health status for not only the life satisfaction variable, but for all of the outcome variables under study. In this study, gender (female), marital status (in a relationship), income level (middle and high levels) and self-rated health (good or very good) increased the odds for experiencing subjective well-being in later life. However, previous studies related to life satisfaction suggest that life evaluations seem to be more sensitive to social circumstances and more often associated with external life domains than other subjective well-being dimensions (Carlquist et al., 2017). Thus, in future research it would be essential to further the evidence on the social gradient and the social circumstances of the individual in particular and their influences on the links between well-being and internet use.

Furthermore, previous research suggests that the framing of life satisfaction questions may also lead to slightly different conclusions regarding associations between subjective well-being and various socio-demographic variables (Jovanović and Lazić, 2020). Thus, it is important to bear in mind that the life satisfaction proxy used within this study differed somewhat from the other two included proxies for subjective well-being. These methodological concerns are further elaborated on in the section “Study strengths and limitations.”

In addition, the cultural context, norms, linguistic and semantic factors also seem to matter and influence our understanding of concepts and terms related to subjective well-being. For instance, a study found that in a Norwegian context, satisfaction was considered to be a more hedonic concept than happiness (Carlquist et al., 2017) and in Denmark (Lolle and Andersen, 2016) the Danish word for happiness referred to something stronger than the English concept–something beyond momentary positive feelings. Danish university students rated their life satisfaction higher when responding to questions posed in Danish than when facing the English concepts (Lolle and Andersen, 2016). Accordingly, it might be that happiness was not an optimal proxy for the hedonic dimension of subjective well-being, nor was life satisfaction maybe not the optimal proxy for the evaluative dimension. Additionally, results from large surveys such as the World Happiness Report display high ratings of life satisfaction among respondents from the Nordic countries, and likewise the present study findings report a larger number of people being satisfied with life than experiencing meaningfulness or happiness. One explanation of the high life satisfaction rates might be connected to the fact that satisfaction (or the Swedish and Finnish words “nöjd” and “tyytyväinen”) might be associated with gratefulness and the norm of modesty (Law of Jante), not expecting or demanding too much, which applies especially to the older generations in the Nordic countries. On the other hand, few people like to regard themselves as dissatisfied with their lives (Huppert, 2014) and there is evidence of overall high well-being rates among older adults living in Western countries. The high levels of well-being seem to be present regardless of functional decline and health problems (Hansen and Slagsvold, 2012), which is sometimes referred to as the aging paradox of well-being and the U-shaped relationship between age and well-being (Blanchflower and Oswald, 2008). However, studies also show declines in levels of well-being among the oldest-olds, Hansen and Slagsvold (2012) and Bauer et al. (2017) argued that some of the dimensions of subjective well-being remain stable in older age, whereas other seem to decline. Thus, what we put into the concept of subjective well-being and associate with the different dimensions seems to also be dependent on cultural and linguistic contexts of the study.

Based on the study findings, what we use the internet for as well as how we conceptualize and measure subjective well-being in studies within this growing research field seem to matter when it comes to the association between subjective well-being and internet use in later life. However, more research is needed to evaluate the relationship between different dimensions of subjective well-being and internet activities.

### Study Strengths and Limitations

Some of the questions and issues raised regarding subjective well-being and internet use in the discussion section can probably be connected to and answered by the methodological limitations of the study at hand. However, the chief objective of the present study was to draw attention to the complexity and multi-dimensionality of both internet use and subjective well-being in order to deepen our understanding of experienced well-being among older adults in a digitized world. As the study is more concept- and theory-driven than epidemiological, some of its methodological limitations might be acceptable and some might even be compensated by the study’s strengths based on large-scale, high-quality survey data, combining two Nordic geographical regions, and covering various groups within the heterogeneous group of older adults.

One of the main weaknesses of this study is its cross-sectional design. Therefore, it is not possible to make statements based on the study findings regarding the causal relation between subjective well-being and internet use. Earlier studies highlight that internet use is more common among resourceful older adults (Hajek and König, 2019) and it is therefore likely that older adults with higher levels of meaningfulness and happiness are more prone to use the internet and internet-based services connected to leisure and entertainment.

Limitations connected to the variables used in the analyses are also worth mentioning. The dataset that this study is based on was collected within a larger Nordic research project (the GERDA project). Using secondary data comes with challenges, such as the fact that the researchers have a certain predetermined number of items and scales available for use that cannot be tailored to the aim of the individual studies using the collected data. However, using secondary data is also efficient and these kinds of larger survey datasets are often considered to have higher validity compared to smaller ones. Additionally, from a research ethical perspective the use of large-scale datasets can also be justified, as the collection of data requires active participation of the respondents and this can also be experienced as challenging for some older persons.

The instruments for assessing meaningfulness and happiness were based on one-item questions with five answer options, which were dichotomized. The focus on one-item proxies for subjective well-being can be considered an additional methodological issue, which might influence the current findings. A risk with using one-item questions when studying multifaceted and somewhat vague concepts such as happiness, meaningfulness and life satisfaction, is that the informants might have interpreted the terms differently and perhaps also as questions regarding the same thing. Capturing nuances between the subjective well-being concepts can be challenging for lay persons (Brülde and Fors, 2014). However, using vague concepts without presenting any definitions opens up the opportunity for the respondents to define the concepts in the way they understand them (Baumeister et al., 2013). Additionally, multiple item-scales often contain items that tap into broad areas, which might slightly differ from the way in which respondents might understand the concepts (Abdel-Khalek, 2006). Other studies have successfully used and tested similar one-item proxies for subjective well-being as used in this study (e.g., happiness: Abdel-Khalek, 2006; meaningfulness: Lampinen et al., 2006; life satisfaction: Cheung and Lucas, 2014; Jovanović and Lazić, 2020) and a recent review identifying and appraising existing instruments to evaluate mental well-being in old age (Martín-María et al., 2021), failed to identify any existing instrument measuring evaluative and experienced well-being (e.g., experienced meaning) among older adults, due to being of poor quality or having limited usability owing to the lack of language translations. Therefore, the choice of variables in the study at hand might be justified.

The life-satisfaction variable was of somewhat different character than the proxies used for meaningfulness and happiness, as the variable was created from a multiple-item scale with dichotomized answer options. There is a risk that important nuances of life satisfaction might have been lost in the analysis. When looking at other studies focusing on internet activities and life satisfaction, similar one item questions as used within this study have been applied, however, often combined with five or four-point answering scales instead of a forced dichotomized answering option as in this study. The aforementioned research connected to life satisfaction and different internet activities (Lissitsa and Chachashvili-Bolotin, 2016; Lifshitz et al., 2018; Gaia et al., 2021) applied either the Satisfaction with Life Scale (developed by Diener, 1985) or a one-item question measuring overall life satisfaction with response options ranging from 1–4 to 1–5. Hence, the loss of nuances might have influenced the study results regarding internet use and life satisfaction in this study.

Moreover, it is also worth mentioning the artificial grouping of the study sample (the somewhat rough cut-off points used in the analyses) might have resulted in a loss of nuances of some of the variables during the recoding process. On the other hand, the focus of this study was on categorical variables and not dividing the sample into groups (or using too many groups) could have led to a loss of statistical power (Sperandei, 2014).

Finally, it is important to consider that older adults with more severe health problems might not have completed the survey (due to the large number of questions) to the same extent as the older adults with better health status might have done. This might have resulted in larger numbers of persons being happy, perceiving their lives as meaningful, being satisfied with their lives as well as persons using the internet within the study sample than among the older population overall.

## Conclusion

The study findings emphasize that socio-environmental factors such as internet use should not be overlooked in studies exploring subjective well-being in later life, especially not in a society where digital technology and online services are being highlighted in strategies related to promoting health and well-being in later life (Nordic Welfare Centre, 2020). However, internet use needs to be conceptualized as a phenomenon beyond infrastructure and access–viewing older adults as agents choosing what they are doing (activities) or what and why they are using (content/purpose) the internet for. There is also a need for further studies on the complexity and multidimensionality of the subjective well-being concept in relation to internet use since internet use and internet activities displayed various associations to the subjective well-being proxies studied.

## Data Availability Statement

The datasets presented in this article are not readily available because the data are part of a larger project conducted in 2016. Requests to access the datasets should be directed to EV, emilia.viklund@abo.fi.

## Ethics Statement

Ethical review and approval was not required for the current study in accordance with the local legislation and institutional requirements. The survey data collection that the study at hand is based upon were reviewed and approved by the Regional Ethical Review Board in Umeå, Sweden (05/084 and 2016/367- 1212 32). The patients/participants provided their written informed consent to participate in this study.

## Author Contributions

EV formulated the aim of the study, conducted the statistical analyses, and was responsible for the initial draft of the manuscript, as well as for the revision and editing of the numerous versions of the manuscript along the process prior to submission. AF formulated the aim of the study, checked and discussed the results of the statistical analyses, and critically reviewed all the versions of the manuscript - from the initial draft to the final version. Both authors contributed equally to the final version of the manuscript and approved the submitted version.

## Conflict of Interest

The authors declare that the research was conducted in the absence of any commercial or financial relationships that could be construed as a potential conflict of interest.

## Publisher’s Note

All claims expressed in this article are solely those of the authors and do not necessarily represent those of their affiliated organizations, or those of the publisher, the editors and the reviewers. Any product that may be evaluated in this article, or claim that may be made by its manufacturer, is not guaranteed or endorsed by the publisher.
